# Markers of Endogenous Desaturase Activity and Risk of Coronary Heart Disease in the CAREMA Cohort Study

**DOI:** 10.1371/journal.pone.0041681

**Published:** 2012-07-23

**Authors:** Yingchang Lu, Anika Vaarhorst, Audrey H. H. Merry, Martijn E. T. Dollé, Robert Hovenier, Sandra Imholz, Leo J. Schouten, Bastiaan T. Heijmans, Michael Müller, P. Eline Slagboom, Piet A. van den Brandt, Anton P. M. Gorgels, Jolanda M. A. Boer, Edith J. M. Feskens

**Affiliations:** 1 Division of Human Nutrition, Wageningen University and Research Center, Wageningen, The Netherlands; 2 National Institute for Public Health and the Environment (RIVM), Bilthoven, The Netherlands; 3 Department of Molecular Epidemiology, Leiden University Medical Center, Leiden, The Netherlands; 4 Department of Epidemiology, CAPHRI School for Public Health and Primary Care, Maastricht University, Maastricht, The Netherlands; 5 Department of Epidemiology, GROW School of Oncology and Developmental Biology, Maastricht University, Maastricht, The Netherlands; 6 Department of Cardiology, University Hospital Maastricht, Maastricht, The Netherlands; University of Oxford, United Kingdom

## Abstract

**Background:**

Intakes of n-3 polyunsaturated fatty acids (PUFAs), especially EPA (C20∶5n-3) and DHA (C22∶6n-3), are known to prevent fatal coronary heart disease (CHD). The effects of n-6 PUFAs including arachidonic acid (C20∶4n-6), however, remain unclear. δ-5 and δ-6 desaturases are rate-limiting enzymes for synthesizing long-chain n-3 and n-6 PUFAs. C20∶4n-6 to C20∶3n-6 and C18∶3n-6 to C18∶2n-6 ratios are markers of endogenous δ-5 and δ-6 desaturase activities, but have never been studied in relation to incident CHD. Therefore, the aim of this study was to investigate the relation between these ratios as well as genotypes of *FADS1* rs174547 and CHD incidence.

**Methods:**

We applied a case-cohort design within the CAREMA cohort, a large prospective study among the general Dutch population followed up for a median of 12.1 years. Fatty acid profile in plasma cholesteryl esters and *FADS1* genotype at baseline were measured in a random subcohort (n = 1323) and incident CHD cases (n = 537). Main outcome measures were hazard ratios (HRs) of incident CHD adjusted for major CHD risk factors.

**Results:**

The AA genotype of rs174547 was associated with increased plasma levels of C204n-6, C20∶5n-3 and C22∶6n-3 and increased δ-5 and δ-6 desaturase activities, but not with CHD risk. In multivariable adjusted models, high baseline δ-5 desaturase activity was associated with reduced CHD risk (*P* for trend = 0.02), especially among those carrying the high desaturase activity genotype (AA): HR (95% CI) = 0.35 (0.15–0.81) for comparing the extreme quintiles. High plasma DHA levels were also associated with reduced CHD risk.

**Conclusion:**

In this prospective cohort study, we observed a reduced CHD risk with an increased C20∶4n-6 to C20∶3n-6 ratio, suggesting that δ-5 desaturase activity plays a role in CHD etiology. This should be investigated further in other independent studies.

## Introduction

Polyunsaturated fatty acids (PUFAs) are generally believed to reduce coronary heart disease (CHD) risk [1], [2], [3], [4]. Intakes of n-3 PUFAs, especially eicosapentaenoic acid (EPA, C20∶5n-3) and docosahexaenoic acid (DHA, C22∶6n-3) present in fish oil, are confirmed to prevent fatal CHD and sudden cardiac death in both observational studies and large-scale randomized controlled trials (RCTs) [1], [3]. However, direct evidence for the preventive effect of n-3 PUFAs on non-fatal CHD was only recently observed in some, but not all, large-scale RCTs [5], [6], [7]. The replacement of saturated fatty acids by n-6 PUFAs protected against incident CHD in a recent meta-analysis including 8 RCTs [8]. As some of these RCTs also included n-3 PUFAs in addition to n-6 PUFAs [2], [8], the effects specific to n-6 PUFAs, however, remain unclear.

**Figure 1:**
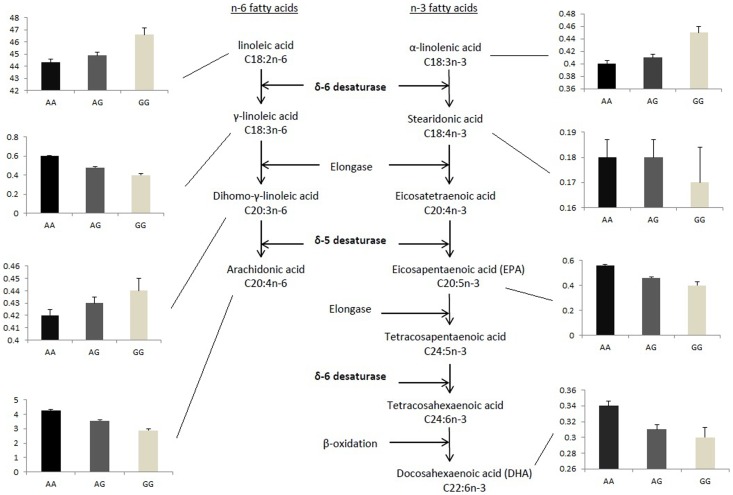
Effect of genotypes of rs174547 on synthesis of PUFAs in the n-3 and n-6 pathways. Measurements of n-3 and n-6 polyunsaturated fatty acid (PUFA) levels in plasma cholesteryl esters in the sub-cohort of CAREMA study (n = 1246, Table 2). The three bars in each of the smaller plots represent levels of fatty acids (%) in individuals who carry AA, AG and GG genotypes of rs174547, respectively.

The fatty acid profile of various biological tissues is often used as a biomarker of dietary fatty acid intake. Adipose tissue reflects the intake of past months to years, while erythrocyte membranes, and plasma or serum phospholipids or cholesteryl esters reflect the intake of several weeks [9], [10], [11]. However, the PUFA profile in biological tissues does not only reflect dietary intake, but is also strongly dependent on the endogenous metabolism of PUFAs [10], [12]. Therefore, PUFA biomarkers in biological tissues mirror the internal PUFA exposure that may be biologically more relevant. Several PUFAs can be endogenously synthesized by a series of alternate desaturation and elongation processes [12], [13]. The δ-5 desaturase and δ-6 desaturase are rate-limiting enzymes for synthesizing long-chain n-3 and n-6 PUFAs (Figure 1) [12], [14], [15], [16]. They are encoded by the *FADS1* and *FADS2* genes on chromosome 11 (11q12–13.1), respectively [12], [17]. Potential functional genetic variants in these genes have been identified including rs174547 [18], and confirmed in recent genome-wide association studies [19], [20], [21]. They have an impact on *FADS1* mRNA abundance [22], [23], [24], [25], [26], and, as a result, on desaturase activity, plasma PUFA levels, and endogenous PUFA pools [18], [19], [20], [21], [26], [27], [28], [29]. Since it is impractical to directly assay the enzyme activities of δ-5 and δ-6 desaturase in humans [12], [14], [15], [29], especially in large-scale epidemiological studies, their activities have traditionally been estimated by using PUFA product-to-precursor ratios [11], [21], [27], [28].

Although few prospective cohort studies have investigated PUFA biomarkers in relation to the incidence of CHD [30], the relation with PUFA product-to-precursor ratios as markers of desaturase activities has, to the best of our knowledge, never been evaluated. In this prospective cohort study, we therefore aim to investigate whether C20∶4n-6 to C20∶3n-6 and C18∶3n-6 to C18∶2n-6 ratios, as respective markers of δ-5 and δ-6 desaturase activity, influence CHD risk.

## Materials and Methods

### Study Population

We conducted a case-cohort study within the Monitoring Project on Cardiovascular Disease Risk Factors 1987–1991 [31], one of the two monitoring studies that were included in the Cardiovascular Registry Maastricht (CAREMA) study. The CAREMA study was described in detail before [32], [33]. In total, 12,486 men and women, born between 1927 and 1967 and living in the Maastricht area, participated in the Monitoring Project on Cardiovascular Disease Risk Factors and had given informed consent to retrieve information from the municipal population registries and from the general practitioner and specialist. The Medical Ethics Committee of the Netherlands Organization for Applied Scientific Research (TNO) approved the study protocol and all participants signed an informed consent form.

### Cardiological Follow-up

The cardiologic follow-up has been described before [32]. In brief, 97.6% of the CAREMA members could be found by linking the cohort to the hospital information system of University Hospital Maastricht (UHM). They were linked to the cardiology information system of the Department of Cardiology to obtain information about the occurrence of myocardial infarction (MI), unstable angina pectoris (UAP), coronary artery bypass grafting (CABG), or percutanous transluminal coronary angioplasty surgery (PTCA). For participants who died, the cause of death was obtained from Statistics Netherlands. In addition, the CAREMA cohort was linked to the hospital discharge registry of the UHM to increase the completeness of the cardiologic follow-up. Follow-up ended on 31 December 2003 with a median follow-up of 12.1 yrs (range: 0.0–16.9 yrs).

### Subcohort and Incident CHD Selection for Case-cohort Design

For the present study, participants who were younger than 30 years at baseline (n = 2204), had a history of MI, UAP, CABG, or PTCA before baseline (n = 118), or were lost to follow-up (n = 2) were excluded. Thus, the eligible cohort consisted of 10,164 participants. All 620 participants who developed incident CHD during follow-up (315 MIs, 244 UAPs and 61 CHD deaths) were included in the case-cohort study. From the eligible cohort, 1483 participants were randomly drawn as a subcohort [34]. By randomly selecting a subcohort and using the specific statistics for this type of research design, the results are expected to be extrapolated to the entire cohort without the need of biomarker measurements in the entire cohort [11], [34], [35], [36].

### Risk Factor Determination

At baseline, all participants filled in a questionnaire on life-style characteristics, medical history, and parental history of MI. During a medical examination, information was collected on blood pressure, height, and weight. In addition, non-fasting blood samples were collected using EDTA tubes. The blood was centrifuged for 10 minutes at 1000 rpm and fractioned into blood plasma, white blood cells and erythrocytes and subsequently stored at −20°C. Within three weeks, the plasma samples were transported to the Lipid Reference Laboratory of the University Hospital Dijkzigt (LRL) in Rotterdam where the total and HDL-cholesterol levels were determined using a CHOD-PAP method [37]. The LRL in Rotterdam is a permanent member of the International Cholesterol Reference Method Laboratory Network.

### Fatty Acid Determination

Fatty acids from plasma cholesteryl esters were quantified by gas-liquid chromatography between 2010 and 2011 at the Department of Human Nutrition of Wageningen University. The case and non-case samples were evenly distributed among the different batches and the assay sequence within each batch was random. The solid-phase extraction method was used to separate the cholesteryl ester fraction from total plasma lipid extracts. Fatty acid methyl esters were prepared by incubating isolated cholesteryl esters with acidified methanol. Peak retention times and area percentages of total fatty acids were identified by using known cholesteryl ester standards (mixture of FAME components from Sigma (MO) and NuChek (MN)) and analyzed with the Agilent Technologies ChemStation software (Agilent, Amstelveen, The Netherlands). For certain fatty acids, the values were too low to be reliably detected in some subjects, and “0” was assigned to their values. Interassay coefficients of variance in fatty acids in plasma cholesteryl esters were 1.68% for C16∶0, 1.01% for C18∶2n-6, 1.88% for C20∶4n-6, and 5.02% for C22∶6n-3, respectively. Fatty acid product-to-precursor ratios were calculated, i.e. C20∶4n-6 to C20∶3n-6 to reflect δ-5 desaturase activity, and C18∶3n-6 to C18∶2n-6 to reflect δ-6 desaturase activity (Figure 1). The 20 subjects with a “0” value for C20∶3n-6 were not included in the analyses for the C20∶4n-6 to C20∶3n-6 ratio, reflecting δ-5 desaturase activity. Information on plasma fatty acids was available on 1323 subcohort members and 537 CHD cases.

### DNA Extraction and Genotyping

DNA was extracted from the white blood cell fraction (buffy coats), using a standard procedure [38]. The resulting DNA pellet was dissolved in TE buffer and DNA concentrations were determined using the Nanodrop ND1000 Spectrophotometer. The single nucleotide polymorphism (SNP) of rs174547 in the *FADS1* gene was selected based on its association with blood cholesterol and triglyceride levels in a genome-wide association study [23]. This SNP is in high linkage disequilibrium (D′ = 1 and *r* ^2^≥0.8) with several other SNPs around the *FADS1* and *FADS2* gene region, which have an impact on mRNA abundance of *FADS1* [22], [23], [24], [25], desaturase activity, plasma PUFA levels, and endogenous PUFA pools [18], [19], [20], [21], [26], [27], [28], [29]. Rs174547 was genotyped entirely independent of case and non-case status using the iPLEX Gold chemistry of Sequenom’s MassARRAY platform (San Diego, CA) at the Leiden University Medical Center. Sequenom’s MassARRAY® Assay Design 3.1 Software was used for SNP assay design, and Sequenom’s SpectroTyper 4.0 software was used to call genotypes automatically, followed by manual review. The total genotyping success rate was 93%. Among the subjects who were measured for plasma fatty acid levels, information on rs174547 genotype was available for 1246 subcohort members and 492 CHD cases. The genotype distribution was consistent with Hardy-Weinberg equilibrium expectations.

### Statistical Analysis

Generalized linear models adjusted for age and sex were used to study the relations of rs174547 genotypes with PUFAs and PUFA ratios. Cox proportional hazards models adapted for the case-cohort design according to the Prentice’s method [35] were used to calculate hazard ratios (HRs) as measures for relative risk [36]. All the major predictors satisfied the proportional hazard assumption (data not shown). We estimated hazard ratios for quintiles of fatty acids (expressed as the percentage of total fatty acids present in the chromatogram) and ratios of C20∶4n-6 to C20∶3n-6 and C18∶3n-6 to C18∶2n-6 based on subcohort distributions, and the respective lowest quintile was used as reference. The base models included age and sex. Additional models were further adjusted for covariates from the Third Report of the National Cholesterol Education Program Expert Panel on Detection, Evaluation, and Treatment of High Blood Cholesterol in Adults (ATP III) risk score based on the Framingham cohort (current smoking, systolic blood pressure, hypertensive medication use, total and HDL cholesterol levels) with the addition of a history of diabetes [39]. The models were also further adjusted for the total percentage of n-3 PUFAs or n-6 PUFAs in plasma cholesteryl esters where necessary. Additional covariates studied were parental history of MI, alcohol use and physical activity. The significance of a linear trend across quintiles of fatty acids and ratios of C20∶4n-6 to C20∶3n-6 and C18∶3n-6 to C18∶2n-6 was examined by including the exposure as a continuous variable in the model. Potential interactions between continuous ratios of C20∶4n-6 to C20∶3n-6 and C18∶3n-6 to C18∶2n-6 and dichotomized rs174547 genotype (homozygous major allele carriers vs. minor allele carriers) were tested by including interaction terms into the model. Statistical significance was considered to be met with a *P* value <0.05 and all testing was 2-sided. All statistical analyses were performed with SAS version 9.1 software (SAS Institute, Cary, NC).

## Results

The general characteristics of the study population by subcohort-case status are shown in Table 1. As expected, cases were older, more frequently male, had higher blood pressure and total cholesterol levels, lower HDL cholesterol levels, smoked more often, and more often reported to have diabetes and a parental history of MI.

**Table 1:** Baseline characteristics of sub-cohort subjects and cases of incident coronary heart disease in the CAREMA cohort study^1^.

	Subcohort (n = 1323)^2^	Cases (n = 537)	Crude HR (95% CI)^3^	Adjusted HR (95% CI)^4^
Age (y)	45.2±8.5	49.7±7.3	1.07 (1.06–1.09)	1.05 (1.04–1.07)
Male sex	608 (46.0%)	392 (73.0%)	3.34 (2.69–4.15)	2.22 (1.66–2.99)
Total cholesterol (mmol/L)	5.7±1.1	6.4±1.2	1.71 (1.56–1.87)	1.42 (1.26–1.60)
HDL cholesterol (mmol/L)	1.2±0.3	1.0±0.2	0.04 (0.03–0.06)	0.09 (0.05–0.16)
Systolic blood pressure (mmHg)	119.2±14.9	128.0±16.9	1.03 (1.02–1.04)	1.02 (1.01–1.03)
Hypertensive medication use	67 (5.1%)	58 (10.8%)	2.34 (1.63–3.35)	1.27 (0.79–2.05)
Diabetes mellitus	13 (1.0%)	20 (3.7%)	5.33 (2.74–10.36)	2.83 (1.39–5.78)
Current smoking	551 (41.8%)	304 (56.7%)	1.81 (1.49–2.21)	1.72 (1.33–2.22)
Parental history of MI	452 (34.3%)	228 (42.5%)	1.40 (1.14–1.71)	1.51 (1.16–1.95)

1 Data are expressed as mean ± SD or n (%) unless otherwise indicated. HDL: high-density lipoprotein; MI: myocardial infarction; and HR (95% CI): hazard ratio and 95% confidence interval.

2 Including 84 cases.

3 Hazard ratios were calculated per unit increase in total cholesterol, HDL cholesterol, and systolic blood pressure, and for the presence of the categorical traits.

4 All variables were added into one multivariable Cox proportional hazards model.

Carrying the minor G allele of rs174547 was associated with higher levels of substrates for desaturases (C18∶2n-6, C20∶3n-6, and C18∶3n-3) and lower levels of products from desaturases (C18∶3n-6, C20∶4n-6, C20∶5n-3, and C22∶6n-3) in the plasma cholesteryl esters. Consequently, lower C20∶4n-6 to C20∶3n-6 and C18∶3n-6 to C18∶2n-6 ratios, as markers of δ-5 and δ-6 desaturase activity, respectively, were observed in carriers of the G allele as compared to those with the AA genotype (Table 2 and Figure 1).

**Table 2:** Association of rs174547 in *FADS1* with baseline PUFAs in plasma cholesteryl esters and desaturase activities in the sub-cohort (n = 1246)^1^.

PUFA	*Rs174547*			*P* value^2^
	AA (545)	AG (569)	GG (132)	
*n-6 PUFA*				
C18∶2n-6 (%)	44.30±0.27^2^	44.88±0.26	46.60±0.54	7.48×10^−4^
C18∶3n-6 (%)	0.60±0.009	0.48±0.009	0.40±0.019	6.87×10^−28^
C20∶3n-6 (%)	0.42±0.005	0.43±0.005	0.44±0.010	0.051
C20∶4n-6 (%)	4.29±0.05	3.56±0.05	2.89±0.09	3.92×10^−46^
*n-3 PUFA*				
C18∶3n-3 (%)	0.40±0.005	0.41±0.005	0.45±0.010	3.28×10^−4^
C18∶4n-3 (%)^3^	0.18±0.007	0.18±0.007	0.17±0.014	0.708
C20∶5n-3 (%)	0.56±0.01	0.46±0.01	0.40±0.03	8.71×10^−8^
C22∶6n-3 (%)	0.34±0.006	0.31±0.006	0.30±0.013	0.005
δ-5^4^	10.65±0.09	8.59±0.09	6.86±0.19	6.40×10^−85^
δ-6^4^	0.014±0.0002	0.011±0.0002	0.009±0.0005	2.51×10^−27^

1 77 subjects in the subcohort had missing values for rs174547. PUFAs: polyunsaturated fatty acids.

2 General linear models were used, and all values are mean ± SEM, adjusted for age and sex.

3 Only few subjects were successfully measured (AA = 161, AG = 185, and GG = 42).

4 δ-5 and δ-6 desaturase activities were assessed by the ratio of C20∶4n-6 to C20∶3n-6 and C18∶3n-6 to C18∶2n-6 in plasma cholesteryl esters, respectively.

A high baseline C20∶4n-6 to C20∶3n-6 ratio was associated with reduced CHD risk (Table 3). A 30% reduction in CHD risk was observed among the participants in the second, third, fourth and fifth quintile of C20∶4n-6 to C20∶3n-6 ratio compared with those in the first quintile after adjustment for age, sex, systolic blood pressure, hypertensive medication use, current smoking, diabetes, total cholesterol, and high-density lipoprotein cholesterol (*P* for trend = 0.02). Although the statistical interaction between rs174547 and δ-5 desaturase activity was not significant (*P* = 0.56), the protective effect of high δ-5 desaturase activity was mainly confined to subjects with the AA genotype (Table S1). In this group, the effect was stronger with a 65% risk reduction for the subjects in the fifth quintile compared with the first quintile (*P* for trend = 0.02). Rs174547 itself was not associated with CHD risk, the age- and sex-adjusted HR per G-allele being 0.99 (95% CI 0.84–1.16, Table S2).

**Table 3:** Association between baseline δ-5 and δ-6 desaturase activity and incident coronary heart disease (CHD).

	Quintile of δ-5 desaturase activity^1^					*P* value for trend^2^
	First (6.45)	Second (7.93)	Third (9.07)	Fourth (10.32)	Fifth (12.52)	
Incident CHD, n	155	117	94	93	67	
Model 1^3^	1	0.70 (0.51–0.97)	0.60 (0.42–0.83)	0.60 (0.43–0.83)	0.49 (0.34–0.70)	<0.0001
Model 2^4^	1	0.75 (0.54–1.06)	0.66 (0.46–0.94)	0.57 (0.39–0.82)	0.51 (0.35–0.75)	<0.0001
Model 3^5^	1	0.68 (0.47–0.98)	0.66 (0.45–0.96)	0.69 (0.46–1.01)	0.68 (0.45–1.02)	0.0249
Model 4^6^	1	0.71 (0.49–1.03)	0.70 (0.48–1.04)	0.74 (0.50–1.09)	0.77 (0.50–1.18)	0.1114
	**Quintile of δ-6 desaturase activity** 1					***P*** ** value for trend** 2
	**First (0.0055)**	**Second (0.0084)**	**Third (0.0104)**	**Fourth (0.0132)**	**Fifth (0.019)**	
**Incident CHD, n**	**92**	**99**	**93**	**122**	**131**	
Model 1^3^	1	0.99 (0.69–1.42)	0.87 (0.60–1.25)	1.09 (0.76–1.55)	1.03 (0.73–1.45)	0.606
Model 2^4^	1	1.03 (0.70–1.51)	0.89 (0.61–1.31)	1.07 (0.73–1.58)	0.93 (0.63–1.36)	0.627
Model 3^5^	1	1.07 (0.71–1.63)	0.86 (0.55–1.33)	1.11 (0.73–1.69)	0.96 (0.63–1.47)	0.897

1 δ-5 and δ-6 desaturase activities were assessed by the ratio of C20∶4n-6 to C20∶3n-6 and the ratio of C18∶3n-6 to C18∶2n-6 in plasma cholesteryl esters, respectively and median ratios in each quintile are listed between brackets.

2 From models with desaturase activity included as a continuous variable.

3 Model 1 was adjusted for age and sex.

4 Model 2 was adjusted for age, sex, systolic blood pressure, hypertensive medication use, current smoking, and diabetes.

5 Model 3 was adjusted for all covariates in model 2, total cholesterol, and high-density lipoprotein cholesterol.

6 Model 4 was adjusted for all covariates in model 3 and percentages of C22∶6n-3 (DHA) in plasma cholesteryl esters.

No association was observed between δ-6 desaturase activity and CHD risk (Table 3), also not after stratification by rs174547 genotype (data not shown).

The results for the four n-6 PUFAs that determine δ-5 and δ-6 desaturase activity are shown in Table S3. No associations with CHD were observed for the C20 precursor (C20∶3n-6) and product (C20∶4n-6, arachidonic acid) of δ-5 desaturase (Figure 1), or for the C18 precursor (C18∶2n-6, linoleic acid) and product (C18∶3n-6) of δ-6 desaturase (Figure 1) after adjustment for age, sex, systolic blood pressure, hypertensive medication use, current smoking, diabetes, total cholesterol, and high-density lipoprotein cholesterol (*P* for trend >0.16).

Regarding the n-3 PUFAs affected by desaturases, a significant inverse association was observed between C22∶6n-3 (DHA) and CHD risk. This association became stronger after adjustment for plasma total and HDL cholesterol levels, and the percentages of n-6 PUFA in plasma cholesteryl esters (*P* for trend = 0.027, Table S4). The proportion of plasma C20∶5n-3 (EPA) was not associated with incident CHD (*P* for trend = 0.724, Table S4). No association was observed between C18∶3n-3 (α-linolenic acid) and CHD risk (data not shown). To explore whether there is any independent effect of C20∶4n-6 to C20∶3n-6 ratio on CHD beyond DHA, we additionally adjusted the models in Table 3 for percentages of DHA. The association between C20∶4n-6 to C20∶3n-6 ratio and CHD risk attenuated, but remained highly significant, especially among the AA carriers of rs174547 (HR:95% CI = 0.44∶0.19–1.04 for comparing the extreme quintiles, Table S1).

Additional adjustment for parental history of MI, alcohol use or physical activity did not materially change any of the aforementioned associations (data not shown).

## Discussion

In this prospective cohort study, we observed an inverse association between C20∶4n-6 to C20∶3n-6 ratio, as the marker of δ-5 desaturase activity, and incident CHD risk, but no association with C18∶3n-6 to C18∶2n-6 ratio, as the marker of δ-6 desaturase activity. This association was partly mediated by DHA. Furthermore we confirmed associations of rs174547 in the *FADS1* gene with plasma PUFA levels and C20∶4n-6 to C20∶3n-6 ratio [18], [19], [20], [21], [27], [28]. Consistent with the established cardiovascular protective effects of n-3 PUFAs [1], [3], and especially tissue DHA [4], [30], high DHA in plasma cholesteryl esters was associated with a reduced CHD risk. However, no association was observed between arachidonic acid or other n-6 PUFAs related to δ-5 or δ-6 desaturase activity in plasma cholesteryl esters and CHD risk.

Common genetic variants (including rs174547) in the *FADS* gene region have been associated with plasma lipid levels (total, LDL and HDL cholesterol, triglycerides, phospholipids and sphingolipids) [19], [21], [23], [40], [41], glycemic traits (fasting glucose and beta-cell function) [26], and resting heart rate [42] in recent genome-wide association studies. However, none of them have been associated with CHD risk directly [40], [43]. This was also the case in our relatively large prospective study. In contrast, when using the estimated δ-5 desaturase activity based on the fatty acid proportion in plasma cholesteryl esters, we found a significant inverse association with incident CHD. This seems contradictory, as a strong association between rs174547 genotypes and estimated δ-5 desaturase activities was observed. However, the reduced risk was already observed with relatively low δ-5 desaturase activities (the second quintile) and remained constant over the following quintiles. Therefore, the majority of the participants with the GG genotype of rs174547 might have sufficient δ-5 desaturase activity to protect them from CHD. This might explain why no association between rs174547 genotypes and CHD risk was found. Both rs174547 genotypes and C20∶4n-6 to C20∶3n-6 ratio reflect endogenous δ-5 desaturase activity, but from two different perspectives. The former can be regarded as the desaturase effect conferred by a single common genetic variant in the *FADS1* gene [20], [26], [27], [28], [29], and the latter as an approximate estimation of full desaturase activity [21], [27], [28]. Their combination might provide the most accurate estimate of δ-5 desaturase activity. This might explain the stronger CHD risk reduction with high δ-5 desaturase activity in the subjects who inherited the AA genotype.

The exact biological mechanisms that link δ-5 desaturase activity with CHD risk are still not well understood. Arachidonic acid, EPA, and DHA are currently considered to be potentially involved directly in the pathogenesis of CHD through thrombotic, inflammatory, arrhythmic and/or lipid regulatory pathways [1], [3], [12], [13], [44], [45], [46]. δ-5 Desaturase is the key enzyme synthesizing these PUFAs, while δ-6 desaturase is important at the beginning of the n-3 and n-6 PUFA synthetic pathways [14], [15]. Therefore, it is biologically plausible that CHD risk could be influenced by δ-5 desaturase activity, but not by δ-6 desaturase activity [12], [13] as was shown in our data. The non-significance of δ-6 desaturase activity on CHD risk is perhaps, also compatible with the reported normal viability and life span of δ-6 desaturase knockout mice [47]. Increased δ-5 desaturase activity might contribute to the intracellular increase of EPA and especially arachidonic acid levels [16]. In non-fish eating populations, arachidonic acid is the predominant tissue very-long-chain PUFA, reaching 80% of the total very-long-chain PUFA [30], [44]. Despite the potential pro-coagulant and pro-inflammatory effects of increased exposures to arachidonic acid and its derived eicosanoid metabolites [2], [13], [44], [45], [46], [48], [49], there is no evidence of increased CHD risk with ≈ 5–7 times habitual arachidonic acid intake based on short-term small-scale controlled feeding studies [2], [50], [51], [52], [53], [54]. Tissue arachidonic acid levels are generally not associated with CHD risk [30]. This was supported by our finding based on the fatty acid profile in plasma cholesteryl esters, which suggests that arachidonic acid does not mediate the observed association between C20∶4n-6 to C20∶3n-6 ratio, as the marker of δ-5 desaturase activity, and CHD risk.

Increased δ-5 desaturase activity (C20∶4n-6 to C20∶3n-6 ratio) was associated with increased plasma levels of EPA and DHA. Our results showed that a possible protective effect of increased δ-5 desaturase activity on CHD may partly be mediated by increased endogenous exposure to DHA. The observation that increased DHA levels associated with increased δ-5 desaturase activity protect against CHD is consistent with the established cardiovascular protective effect of increased n-3 PUFA exposure (EPA and/or DHA) [1], [3]. Accumulating evidence from observational studies suggests that DHA might be more protective for CHD than EPA [4], [30], which is consistent with our findings. However, EPA and DHA are usually correlated with each other in tissues, and their potential effects cannot be easily discerned. More research on this issue is therefore warranted. In addition to blood triglyceride lowering and HDL cholesterol increasing effects of EPA and DHA, n-3 PUFAs have long been observed to have anti-thrombotic, anti-inflammatory, anti-arrhythmic, and blood pressure-lowering effects in humans even though the underlying mechanisms for these effects are incompletely understood [1], [3], [12], [13], [46]. Interestingly, the protective effects on fatal CHD and sudden cardiac death have been shown to level off with a modest intake of EPA and/or DHA (250 mg/day), and little additional benefit was observed with higher intakes [1]. This is also consistent with our results for C20∶4n-6 to C20∶3n-6 ratio as the marker of δ-5 desaturase activity. Nevertheless, there might be other unidentified pleiotropic cardiovascular protective effects of increased δ-5 desaturase activity. For example, these desaturases are also significantly expressed in immune cells [55], [56] that play important roles in atherosclerotic CHD progression.

Our results should be interpreted in the context of several limitations. First, our analyses were based on a single baseline measurement of fatty acid levels in plasma cholesteryl esters that may not accurately reflect long-term fatty acid exposures. However, we did detect the established protective effect of DHA against CHD [1], [3], [4], [12], [13], [30]. Second, we estimated δ-5 and δ-6 desaturase activities based on n-6 PUFAs, while δ-5 and δ-6 desaturases are not only involved in n-6 PUFA conversion, but also in n-3 PUFA conversion. However, in comparison to n-6 PUFA conversion, the amount of n-3 PUFA conversion is relatively small [16], which should not have affected our results. Third, other potential unmeasured environmental or physiological factors could have confounded the observed associations. However, the relatively large magnitude of the protective effect of increased δ-5 desaturase activity relative to the effect of other risk factors for CHD makes confounding with other unknown risk factors unlikely. Finally, our models that included total and HDL cholesterol may have been over-adjusted, as these are probably intermediates in the metabolic pathway between desaturase and CHD risk (Note S1).

In conclusion, in this prospective cohort study, we observed a reduced CHD risk with increased C20∶4n-6 to C20∶3n-6 ratio that was partly mediated by DHA. These results suggest that δ-5 desaturase activity plays a role in protecting us from CHD.

## Supporting Information

Table S1 Association between baseline δ-5 desaturase activity and incident coronary heart disease according to rs174547 genotypes.
(DOCX)

Table S2 Association of rs174547 with incident coronary heart disease (CHD) risk.
(DOCX)

Table S3 Association between baseline n-6 PUFA in plasma cholesteryl esters (precursors and products of δ5- or δ6-desaturase) and incident coronary heart disease (CHD).
(DOCX)

Table S4 Association of baseline C20∶5n-3 (EPA) and C22∶6n-3 (DHA) in plasma cholesteryl esters with incident coronary heart disease (CHD).
(DOCX)

Note S1 Analysis of intermediate factors of coronary heart disease (CHD).
(DOCX)
