# Supplementary material for: Markers of Endogenous Desaturase Activity and Risk of Coronary Heart Disease in the CAREMA Cohort Study
Source: PLoS One. 2012 Jul 23;7(7):e41681. doi: 10.1371/journal.pone.0041681 (PMC3402436; doi:10.1371/journal.pone.0041681)
Supplement: Table S1 — Association between baseline δ-5 desaturase activity and incident coronary heart disease according to rs174547 genotypes. (DOCX) [file pone.0041681.s001.docx]

**Table S1.** Association between baseline δ-5 desaturase activity and incident coronary heart disease according to rs174547 genotypes

| *rs174547*  (# cases) | Quintile of δ-5 desaturase activity*^1^* | | | | | *P* value for trend*^2^* | |
| --- | --- | --- | --- | --- | --- | --- | --- |
|  | First | Second | Third | Fourth | Fifth | |  |
| AA  (n = 205) | 1*^3^* | 0.61 (0.27-1.41) | 0.35 (0.15-0.79) | 0.36 (0.16-0.80) | 0.25 (0.11-0.54) | | <0.0001 |
|  | 1*^4^* | 0.60 (0.25-1.43) | 0.35 (0.15-0.83) | 0.48 (0.21-1.11) | 0.35 (0.15-0.81) | | 0.022 |
|  | 1*^5^* | 0.63 (0.26-1.53) | 0.40 (0.17-0.97) | 0.55 (0.24-1.27) | 0.44 (0.19-1.04) | | 0.087 |
| AG/GG  (n = 276) | 1*^3^* | 0.69 (0.46-1.03) | 0.62 (0.38-1.00) | 0.54 (0.31-0.93) | 1.15 (0.57-2.33) | | 0.027*^6^* |
|  | 1*^4^* | 0.62 (0.39-0.98) | 0.71 (0.42-1.21) | 0.72 (0.40-1.30) | 1.64 (0.76-3.53) | | 0.463 |
|  | 1*^5^* | 0.63 (0.40-1.02) | 0.75 (0.44-1.27) | 0.78 (0.43-1.41) | 1.86 (0.84-4.12) | | 0.649 |

*^1^* δ-5 desaturase activity was assessed by the ratio of C20:4n-6 to C20:3n-6 in plasma cholesteryl esters.

*^2^* From models with desaturase activity included as a continuous variable.

*^3^* Model was adjusted for age, sex, systolic blood pressure, hypertensive medication use, current smoking, and diabetes.

*^4^* Model was adjusted for age, sex, systolic blood pressure, hypertensive medication use, current smoking, diabetes, total cholesterol, and high-density lipoprotein cholesterol.

*^5^* Model was adjusted for age, sex, systolic blood pressure, hypertensive medication use, current smoking, diabetes, total cholesterol, high-density lipoprotein cholesterol, and percentages of C22:6n-3 (DHA) in plasma cholesteryl esters.

*^6^* Hazard ratio per unit desaturase activity (95% confidence interval) = 0.89 (0.80-0.99).
