# Supplementary material for: Markers of Endogenous Desaturase Activity and Risk of Coronary Heart Disease in the CAREMA Cohort Study
Source: PLoS One. 2012 Jul 23;7(7):e41681. doi: 10.1371/journal.pone.0041681 (PMC3402436; doi:10.1371/journal.pone.0041681)
Supplement: Table S2 — Association of rs174547 with incident coronary heart disease (CHD) risk. (DOCX) [file pone.0041681.s002.docx]

**Table S2.** Association of rs174547 with incident coronary heart disease (CHD) risk

|  | rs174547 genotype | | | rs174547 G allele*^1^* |
| --- | --- | --- | --- | --- |
|  | *AA* | *AG* | *GG* |  |
| Incident CHD, n | 234 | 259 | 56 | - |
| Model 1*^2^* | 1.0 | 1.00 (0.80-1.26) | 0.96 (0.67-1.37) | 0.99 (0.84-1.16) |
| Model 2*^3^* | 1.0 | 1.15 (0.88-1.49) | 0.91 (0.60-1.39) | 1.02 (0.85-1.22) |

*^1^* The model assumes that each copy of the G allele contributes equally to coronary heart disease risk.

*^2^* Model 1 was adjusted for age and sex.

*^3^* Model 2 was adjusted for age, sex, systolic blood pressure, hypertensive medication use, current smoking, diabetes, total cholesterol, and high-density lipoprotein cholesterol.
