# Supplementary material for: Markers of Endogenous Desaturase Activity and Risk of Coronary Heart Disease in the CAREMA Cohort Study
Source: PLoS One. 2012 Jul 23;7(7):e41681. doi: 10.1371/journal.pone.0041681 (PMC3402436; doi:10.1371/journal.pone.0041681)
Supplement: Table S3 — Association between baseline n-6 PUFA in plasma cholesteryl esters (precursors and products of δ5- or δ6-desaturase) and incident coronary heart disease (CHD). (DOCX) [file pone.0041681.s003.docx]

**Table S3.** Association between baseline n-6 PUFA in plasma cholesteryl esters (precursors and products of δ5- or δ6-desaturase) and incident coronary heart disease (CHD)

|  | Quintile of C18:2n-6 (linoleic acid) | | | | | *P* value for trend*^1^* |
| --- | --- | --- | --- | --- | --- | --- |
|  | First | Second | Third | Fourth | Fifth |  |
| Incident CHD, n | 127 | 125 | 102 | 97 | 86 |  |
| Model 1*^2^* | 1 | 1.11 (0.80-1.54) | 1.02 (0.72-1.44) | 1.10 (0.78-1.56) | 0.80 (0.57-1.13) | 0.247 |
| Model 2*^3^* | 1 | 1.16 (0.81-1.66) | 1.27 (0.88-1.83) | 1.30 (0.89-1.89) | 0.91 (0.62-1.32) | 0.861 |
| Model 3*^4^* | 1 | 1.09 (0.74-1.60) | 1.34 (0.91-1.96) | 1.38 (0.92-2.09) | 1.01 (0.68-1.48) | 0.395 |
| Model 4*^5^* | 1 | 1.09 (0.74-1.61) | 1.34 (0.91-1.96) | 1.39 (0.92-2.09) | 1.01 (0.69-1.49) | 0.389 |
|  | Quintile of C18:3n-6 | | | | | *P* value for trend*^1^* |
|  | First | Second | Third | Fourth | Fifth |  |
| Incident CHD, n | 93 | 109 | 100 | 107 | 128 |  |
| Model 1*^2^* | 1 | 1.05 (0.74-1.50) | 0.89 (0.62-1.28) | 0.92 (0.64-1.32) | 1.00 (0.71-1.41) | 0.706 |
| Model 2*^3^* | 1 | 1.12 (0.76-1.64) | 0.90 (0.61-1.33) | 1.02 (0.69-1.50) | 0.93 (0.64-1.37) | 0.867 |
| Model 3*^4^* | 1 | 1.13 (0.75-1.72) | 0.90 (0.58-1.38) | 1.01 (0.66-1.53) | 1.05 (0.70-1.57) | 0.605 |
| Model 4*^5^* | 1 | 1.14 (0.75-1.73) | 0.90 (0.58-1.39) | 1.02 (0.67-1.56) | 1.06 (0.70-1.61) | 0.536 |
|  | Quintile of C20:3n-6 | | | | | *P* value for trend*^1^* |
|  | First | Second | Third | Fourth | Fifth |  |
| Incident CHD, n | 88 | 80 | 107 | 125 | 137 |  |
| Model 1*^2^* | 1 | 0.98 (0.68-1.43) | 1.14 (0.80-1.64) | 1.29 (0.91-1.82) | 1.39 (0.99-1.96) | 0.024 |
| Model 2*^3^* | 1 | 0.91 (0.61-1.34) | 1.06 (0.73-1.55) | 1.14 (0.78-1.66) | 1.43 (1.00-2.05) | 0.011 |
| Model 3*^4^* | 1 | 0.94 (0.61-1.45) | 1.13 (0.75-1.69) | 1.00 (0.67-1.50) | 1.11 (0.75-1.67) | 0.494 |
| Model 4*^5^* | 1 | 0.95 (0.62-1.47) | 1.15 (0.76-1.73) | 1.03 (0.67-1.57) | 1.14 (0.75-1.74) | 0.420 |
|  | Quintile of C20:4n-6 (arachidonic acid) | | | | | *P* value for trend*^1^* |
|  | First | Second | Third | Fourth | Fifth |  |
| Incident CHD, n | 127 | 107 | 120 | 79 | 104 |  |
| Model 1*^2^* | 1 | 0.89 (0.63-1.24) | 0.96 (0.70-1.34) | 0.65 (0.45-0.92) | 0.76 (0.54-1.06) | 0.031 |
| Model 2*^3^* | 1 | 0.86 (0.60-1.22) | 0.92 (0.65-1.31) | 0.64 (0.43-0.94) | 0.77 (0.53-1.10) | 0.088 |
| Model 3*^4^* | 1 | 1.06 (0.72-1.56) | 1.09 (0.74-1.60) | 0.79 (0.51-1.21) | 0.86 (0.58-1.28) | 0.163 |
| Model 4*^5^* | 1 | 1.04 (0.70-1.55) | 1.07 (0.73-1.58) | 0.76 (0.48-1.20) | 0.83 (0.53-1.28) | 0.150 |

*^1^* From models with fatty acids included as continuous variables.

*^2^* Model 1 is adjusted for age and sex.

*^3^* Model 2 is adjusted for age, sex, systolic blood pressure, hypertensive medication use, current smoking, and diabetes.

*^4^* Model 3 is adjusted for all covariates in model 2, total cholesterol, and high-density lipoprotein cholesterol.

*^5^* Model 4 is adjusted for all covariates in model 3 and baseline n-3 PUFA in plasma cholesteryl esters.
