# Supplementary material for: Markers of Endogenous Desaturase Activity and Risk of Coronary Heart Disease in the CAREMA Cohort Study
Source: PLoS One. 2012 Jul 23;7(7):e41681. doi: 10.1371/journal.pone.0041681 (PMC3402436; doi:10.1371/journal.pone.0041681)
Supplement: Table S4 — Association of baseline C20∶5n-3 (EPA) and C22∶6n-3 (DHA) in plasma cholesteryl esters with incident coronary heart disease (CHD). (DOCX) [file pone.0041681.s004.docx]

**Table S4.** Association of baseline C20:5n-3 (EPA) and C22:6n-3 (DHA) in plasma cholesteryl esters with incident coronary heart disease (CHD)

|  | Quintile of C20:5n-3 (EPA) | | | | | *P* value for trend*^2^* |
| --- | --- | --- | --- | --- | --- | --- |
|  | First  (0.00)*^1^* | Second  (0.28) | Third  (0.38) | Fourth  (0.52) | Fifth  (0.83) |  |
| Incident CHD, n | 96 | 100 | 104 | 114 | 123 |  |
| Model 1*^3^* | 1 | 0.81 (0.57-1.16) | 0.88 (0.62-1.26) | 0.82 (0.57-1.16) | 0.82 (0.58-1.17) | 0.348 |
| Model 2*^4^* | 1 | 0.86 (0.59-1.24) | 0.91 (0.62-1.33) | 0.88 (0.61-1.27) | 0.76 (0.52-1.11) | 0.243 |
| Model 3*^5^* | 1 | 0.90 (0.60-1.34) | 0.90 (0.59-1.39) | 0.79 (0.52-1.20) | 0.89 (0.58-1.35) | 0.733 |
| Model 4*^6^* | 1 | 0.90 (0.60-1.35) | 0.90 (0.59-1.39) | 0.80 (0.52-1.22) | 0.89 (0.58-1.35) | 0.724 |
|  | Quintile of C22:6n-3 (DHA) | | | | | *P* value for trend*^2^* |
|  | First  (0.00)*^1^* | Second  (0.20) | Third  (0.27) | Fourth  (0.34) | Fifth  (0.46) |  |
| Incident CHD, n | 89 | 139 | 93 | 112 | 104 |  |
| Model 1*^3^* | 1 | 1.45 (1.03-2.05) | 0.87 (0.60-1.25) | 1.07 (0.74-1.53) | 0.93 (0.65-1.34) | 0.286 |
| Model 2*^4^* | 1 | 1.49 (1.05-2.13) | 0.84 (0.58-1.24) | 1.13 (0.77-1.67) | 0.86 (0.58-1.29) | 0.268 |
| Model 3*^5^* | 1 | 0.96 (0.65-1.43) | 0.61 (0.41-0.92) | 0.89 (0.59-1.34) | 0.65 (0.42-0.99) | 0.049 |
| Model 4*^6^* | 1 | 0.95 (0.64-1.40) | 0.57 (0.38-0.87) | 0.82 (0.53-1.26) | 0.59 (0.37-0.93) | 0.027 |

*^1^* Median percentages of EPA and DHA in each quintile are listed between brackets.

*^2^* From models with fatty acids included as continuous variables.

*^3^* Model 1 is adjusted for age and sex.

*^4^* Model 2 is adjusted for age, sex, systolic blood pressure, hypertensive medication use, current smoking, and diabetes.

*^5^* Model 3 is adjusted for all covariates in model 2, total cholesterol, and high-density lipoprotein cholesterol.

*^6^* Model 4 is adjusted for all covariates in model 3 and baseline n-6 PUFA in plasma cholesteryl esters.
