# Supplementary material for: Markers of Endogenous Desaturase Activity and Risk of Coronary Heart Disease in the CAREMA Cohort Study
Source: PLoS One. 2012 Jul 23;7(7):e41681. doi: 10.1371/journal.pone.0041681 (PMC3402436; doi:10.1371/journal.pone.0041681)
Supplement: Note S1 — Analysis of intermediate factors of coronary heart disease (CHD). (DOCX) [file pone.0041681.s005.docx]

**Supplementary Note S1**

**Analysis of intermediate factors of coronary heart disease (CHD)**

To explore whether δ-5 desaturase activity might exert its protective effect against CHD through known intermediate risk (or protective) factors of CHD (total and HDL cholesterol levels, systolic blood pressure, diabetes mellitus, EPA and DHA), we regressed these factors against δ-5 desaturase activity with adjustment for age and sex. It seems some of these intermediate factors (HDL cholesterol levels, EPA and DHA) might be involved in the protective effects of δ-5 desaturase activity against CHD risk (See Supplementary Table 5).

Table S5. Association between intermediate risk (protective) factors for coronary heart disease and δ-5 desaturase activity at baseline in a random subcohort (n = 1263).*^1^*

| Covariates | *B* | *P* |
| --- | --- | --- |
| Total cholesterol (mmol/L)*^2^* | -0.021 ± 0.011 | 0.076 |
| HDL cholesterol (mmol/L)*^2^* | 0.020 ± 0.003 | < 0.0001 |
| Systolic blood pressure (mmHg)*^2^* | -0.29 ± 0.17 | 0.078 |
| EPA (%)*^2^* | 0.044 ± 0.004 | < 0.0001 |
| DHA (%)*^2^* | 0.023 ± 0.002 | < 0.0001 |
| Diabetes mellitus (Yes/no)*^3^* | -0.020 ± 0.12 | 0.86 |

*^1^* δ-5 desaturase activity was assessed by the ratio of C20:4n-6 to C20:3n-6 in plasma cholesteryl esters.

*^2^* *B* and *P* are β regression coefficients ± SE and corresponding p values obtained by using a regression model adjusted for age and sex.

*^3^* *B* and *P* are β regression coefficient ± SE and corresponding p value obtained by using a logistic model adjusted for age and sex.
